# Abivertinib synergistically strengthens the anti‐leukemia activity of venetoclax in acute myeloid leukemia in a BTK‐dependent manner

**DOI:** 10.1002/1878-0261.12742

**Published:** 2020-07-03

**Authors:** Shujuan Huang, Chenying Li, Xiang Zhang, Jiajia Pan, Fenglin Li, Yunfei Lv, Jingwen Huang, Qing Ling, Wenle Ye, Shihui Mao, Xin Huang, Jie Jin

**Affiliations:** ^1^ Department of Hematology the First Affiliated Hospital Zhejiang University College of Medicine Hangzhou China; ^2^ Key Laboratory of Hematologic Malignancies, Diagnosis and Treatment, Zhejiang Hangzhou China

**Keywords:** abivertinib, AML, BTK‐dependent, venetoclax

## Abstract

B‐cell lymphoma 2 (BCL‐2), a crucial member of the anti‐apoptotic BCL‐2 family, is frequently dysregulated in cancer and plays an important role in acute myeloid leukemia (AML). Venetoclax is a highly selective BCL‐2 inhibitor that has been approved by the FDA for treating elderly AML patients. However, the emergence of resistance after long‐term treatment emphasizes the need for a deeper understanding of the potential mechanisms of resistance and effective rescue methods. By using RNA‐seq analysis in two human AML cohorts made up of three patients with complete remission and three patients without remission after venetoclax treatment, we identified that upregulation of BTK enabled AML blast resistance to venetoclax. Interestingly, we found that abivertinib, an oral BTK inhibitor, could synergize with venetoclax to inhibit the proliferation of primary AML cells and cell lines. It is worth noting that the combination of the two effectively enhanced the sensitivity of two AML patients (AML#3 and AML#12) to venetoclax. In this study, we demonstrated that combined use of the two drugs can synergistically inhibit the colony‐forming capacity of AML cells, arrest the AML cell cycle in the G0/G1 phase, and inhibit the BCL‐2 anti‐apoptotic family protein, activating the caspase family to induce apoptosis. Mechanistically, knockdown of BTK in AML cell lines impaired the synergistic effect of the two drugs. *In vivo* study showed similar results as those seen *in vitro*. Abivertinib in combination with venetoclax could significantly prolong the survival time and reduce the tumor burden of MV4‐11‐NSG mice compared with those of control and single‐agent groups. Our *in vitro* and *in vivo* studies have shown that the combination of abivertinib and venetoclax may benefit AML patients, especially in patients resistant to venetoclax or those that relapse. New clinical trials will be planned.

## Graphical abstract

Abivertinib could rescue AML insensitive to venetoclax‐involved therapy.

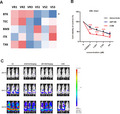

**Abbreviations**
AML: acute myeloid leukemia
BCL‐2: B‐cell lymphoma 2
BTK: Bruton tyrosine kinase
DLBCL: diffuse large cell lymphoma
FCL: follicular cell lymphoma
FLT3‐ITD: FMS‐like tyrosine kinase‐3‐internal tandem duplication mutations
NSG: NOD‐SCID gamma

## 1 Introduction

Acute myeloid leukemia (AML) is a hematopoietic malignancy characterized by the block of differentiation and clonal proliferation of myeloid precursor cells leading to the disruption of normal hematopoiesis [1]. Despite recent developments in diagnosis and therapy, long‐term survival of patients with AML is still low [2]. Clonal amplification of AML blasts typically occurs following a series of somatic mutations in relatively few genes required for transcription, cellular signaling, epigenetic modification, methylation, DNA repair, or other critical cellular processes. All of these mutations provide survival advantages to subpopulations of neoplastic cells [3, 4, 5, 6].

The B‐cell lymphoma 2 (BCL‐2) protein plays an important role in the survival and persistence of AML blasts, as it is a key regulator of the mitochondrial apoptotic pathway [7, 8]. Venetoclax, a potent, selective, oral inhibitor of BCL‐2, has demonstrated clinical activity and a tolerable safety profile in patients with relapsed or refractory AML. However, several recent studies have been reported that patients treated with venetoclax gradually develop drug resistance in the clinical application of AML therapy [9, 10, 11, 12, 13, 14]. There were reports that showed the safety and efficacy of venetoclax with decitabine or azacitidine in clinical trial [7, 15]. Additionally, their result demonstrated the combination of venetoclax‐based therapy was effective and well‐tolerated in elderly patients with AML. However, 30% of AML patients still do not achieve complete remission with the combination therapy or relapse after a short time [15]. Hence, several research groups have worked hard to identify that the upregulation of MCL‐1 is responsible for venetoclax resistance [8, 16, 17, 18, 19, 20, 21, 22]. Most of these studies only focused on the mitochondrial apoptotic pathway.

On the other hand, BTK inhibitors have been widely applied in hematologic malignancies, including mantle cell lymphoma, DLBCL, and FCL [23, 24, 25]. In recent years, their therapeutic effects in AML have also been confirmed [26, 27]. Our earlier studies have shown that a novel BTK inhibitor, abivertinib, can effectively inhibit the progression of AML *in vitro* and *in vivo* in combination with homoharringtonine [28].

As we know, chemo‐free small‐molecule inhibitor therapy is the future trend, but the efficacy of single‐agent therapy is very limited, so the combination of small‐molecule targeted drugs may benefit more AML patients. In this AML study, we considered two drugs, a combination of two oral small‐molecule inhibitors, which have achieved good results. Our *in vitro* and *in vivo* studies have shown that abivertinib and venetoclax work synergistically in treating AML, which implies a promising chemo‐free therapy for elderly AML patients and those who are not tolerant to chemotherapy.

## 2 Materials and methods [28]

### 2.1 Materials

Phosphorylated and total BTK (Tyr223), AKT (Ser473), PI3K (p110α), PLCγ2 (Tyr759), IKK (Ser176/180), NF‐κB (Ser536), CDK2, CDK4, CDK6, caspase‐3, caspase‐8, PARP, Bad, Bax, BCL‐2, BCL‐XL, and GAPDH antibodies were purchased from Cell Signaling Technology (Beverly, MA, USA). A human CD45 antibody was purchased from Abcam (Cambridge, MA, USA). Abivertinib was synthesized by Hangzhou ACEA Pharmaceutical Research Co., Ltd (West Lake District, Hangzhou, China). Venetoclax was purchased from Selleck Chemicals (Houston, TX, USA).

### 2.2 Cell lines and primary cells

MV4‐11 and MOLM‐13, human AML cells harboring FLT3 internal tandem duplication (ITD) mutations, were a kind gift from Professor R. Bhatia (City of Hope National Medical Center, Duarte, CA, USA) and were cultured in Iscove's modified Dulbecco's medium supplemented with 10% FBS. KG‐1 and THP‐1 cell lines were purchased from the Shanghai Cell Bank of the Chinese Academy of Sciences. These cells were cultured in RPMI 1640 medium supplemented with 10% FBS.

### 2.3 Patient material

Bone marrow and peripheral blood samples were obtained from AML patients following written informed consent. Mononuclear cells were isolated by Ficoll‐Hypaque (Sigma‐Aldrich, St. Louis, MO, USA) density gradient centrifugation. Testing for gene mutations (*FLT3‐ITD, NPM1, DMNT3A, IDH1, IDH2*) was performed at the First Affiliated Hospital of Zhejiang University, Hangzhou, China. Cord blood CD34‐positive hematopoietic stem cells were provided by healthy donors. The study protocol was approved by the Ethics Committee of the First Affiliated Hospital of Zhejiang University, China. The study methodologies conformed to the standards set by the Declaration of Helsinki.

### 2.4 Colony‐forming assay

Three percent soft AGAR was diluted to 1% for the lower layer and 0.4% for the upper layer. AML cells were seeded in 6‐well plates (0.5–1 × 10^3^ cells/well) in triplicate and treated with abivertinib (156 nm), venetoclax (50 nm), or their combination for 14 days. Cell colonies were stained with 0.05% crystal violet solution for 30 min and counted.

### 2.5 Cell proliferation assay

Cells were seeded in 96‐well plates (1 × 10^3^–1 × 10^4^ cells/well) in triplicate and treated with different drugs (abivertinib, venetoclax, and COM) for 24 h. Twenty microliters of MTS solution (Promega CellTitre 96, Promega Corporation, Madison, WI, USA) (5 mg·mL^−1^) was added to each well followed by incubation for an additional 4 h at 37 °C. Cell numbers were assessed based on the quantification of formazan by determining the absorbance at 490 nm.

### 2.6 Apoptosis assay

Induction of apoptosis was assessed using an apoptosis detection kit (BD PharMingen, San Diego, CA, USA). After treatment with drugs for 24 or 48 h, the cells were washed twice with PBS, resuspended in binding buffer, and incubated with Annexin V‐FITC and propidium iodide (PI) for 15 min. Apoptotic cells were analyzed by flow cytometry using a FACScan™ flow cytometer (Becton Dickinson, San Diego, CA, USA).

### 2.7 Cell cycle analysis

After treatment with drugs for 24 or 48 h, cells were harvested and fixed overnight with 75% ethanol at 4 °C, followed by two PBS washes and incubation in a buffer containing 50 μg·mL^−1^ PI and 100 μg·mL^−1^ RNase A for 30 min at room temperature. Cell cycle analysis was conducted using a FACScan™ flow cytometer (Becton Dickinson).

### 2.8 Western blot analysis

Cells were lysed in radioimmunoprecipitation buffer (Cell Signaling Technology) on ice for 30 min. The protein concentration of the cellular supernatant was determined using BCA reagent after centrifugation of the cell lysate at 12 000 ***g*** for 15 min at 4 °C. Western blotting was performed after 10% SDS/PAGE (Life Technologies, Carlsbad, CA, USA), with the cellular proteins transferred onto a preactivated PVDF membrane (Millipore, Billerica, MA, USA). The membranes were blocked with 5% nonfat milk for 1 h and incubated with primary antibodies overnight at 4 °C. After incubation with primary antibodies, the blots were washed thrice with TBST buffer, and membranes were incubated with secondary antibodies (Cell Signaling Technology) for 1 h at room temperature. The target proteins were visualized using an ECL detection kit (Amersham, Little Chalfont, UK) and analyzed using image lab™ software (Bio‐Rad Laboratories, Hercules, CA, USA).

### 2.9 RNA interference

The siRNAs against BTK and MCL‐1 were purchased from Invitrogen (Pittsburg, PA, USA) and were transfected into THP‐1 cells using Invitrogen™ Lipofectamine™ RNAiMAX (Fisher Scientific, Pittsburg, PA, USA), according to the manufacturer's instructions. In brief, the cells were seeded at a density of 5 × 10^5^/well in 6‐well plates. About 100 nm siRNA and 5 μL Lipofectamine were mixed in 500 μL Opti‐MEM (Fisher Scientific) and incubated at room temperature for 20 min before adding the transfection mix to cells. Cells were then incubated at 37 °C for 72 h.

### 2.10 RNA extraction and real‐time PCR (qRT–PCR)

Total RNA was extracted from the cells using TRIzol reagent according to manufacturer's instructions. Reverse transcription was performed using the RNA PCR core kit (Life Technologies, Paisley, UK). Quantitative real‐time PCR was carried out using SYBR Green qPCR Master Mix, and GAPDH was used as an internal control. The sequences of the primers were as follows: GAPDH forward, 5′‑GGAGCGAGATCCCTCCAAAAT‑3′, and reverse, 5′‑GGCTGTTGTCATACTTCTCATGG‑3′; BTK forward, 5′‑TCTGAAGCGATCCCAACAGAA‑3′, and reverse, 5′‑TGCACGGTCAAGAGAAACAGG‑3′; and MCL‐1 forward, 5′‑TGCTTCGGAAACTGGACATCA‑3′, and reverse, 5′‑TAGCCACAAAGGCACCAAAAG‑3′.

### 2.11 RNA sequencing

Bone marrow mononuclear cells were collected from six AML patients treated with the ‘VA’ program (BCL‐2 inhibitor venetoclax combined with azacitidine). Among them, three patients had achieved complete remission and three patients had not responded to the therapy. The RNA sequencing was performed by Annuoda Company (Nanjing, China), and the expression levels related to the TEC family were analyzed.

### 2.12 AML xenograft model

NOD‐SCID gamma (NSG) mice were purchased from Shanghai SLRC Laboratory Animal Center. Our animal study was approved by the Ethics Committee for Laboratory Animals of the First Affiliated Hospital, College of Medicine, Zhejiang University (Hangzhou, China), and was conducted in accordance with the National Institutes of Health Guide for the Care and Use of Laboratory Animals. Mice were exposed to a 10/14‐h light**–**dark cycle, kept under normal room temperature, and fed standard pellet food and tap water.

For the MV4‐11‐NSG xenograft model (Biocytogen, Nanjing, China), 2 × 10^6^ MV4‐11 cells were injected into the NSG mice via the tail vein. After 7 days, cell engraftment was assessed by the expression of human CD45 by PBMCs of mice each week. Mice were randomly assigned to four groups according to the expression of human CD45. The four groups were treated as follows: 0.5% methylcellulose (MC, days 8–26, PO), 50 mg·kg^−1^ abivertinib (days 8–26, PO), 75 mg·kg^−1^ venetoclax (days 8–26, PO), or 50 mg·kg^−1^ abivertinib and 75 mg·kg^−1^ venetoclax (administered per the single‐agent groups). The mice were humanely sacrificed after observation of typical leukemic symptoms. PBMC, bone marrow, and spleen samples were harvested for flow cytometry analysis and IHC. Human CD45 antibody was used to identify engraftments.

The study methodologies conformed to the standards set by the Declaration of Helsinki.

### 2.13 Statistical analyses

Data were analyzed using graphpad prism 6.0 software (Shanghai, China). Summary statistics (mean ± SD) are represented with statistical significance assessed using the Mann**–**Whitney test (*P* < 0.05 was considered statistically significant). Survival was analyzed using the Kaplan**–**Meier method and analyzed using a log‐rank test.

## 3 Results

### 3.1 AML cells adaptively resistant to BCL‐2 inhibitor display an increased BTK expression

**Fig. 1:**
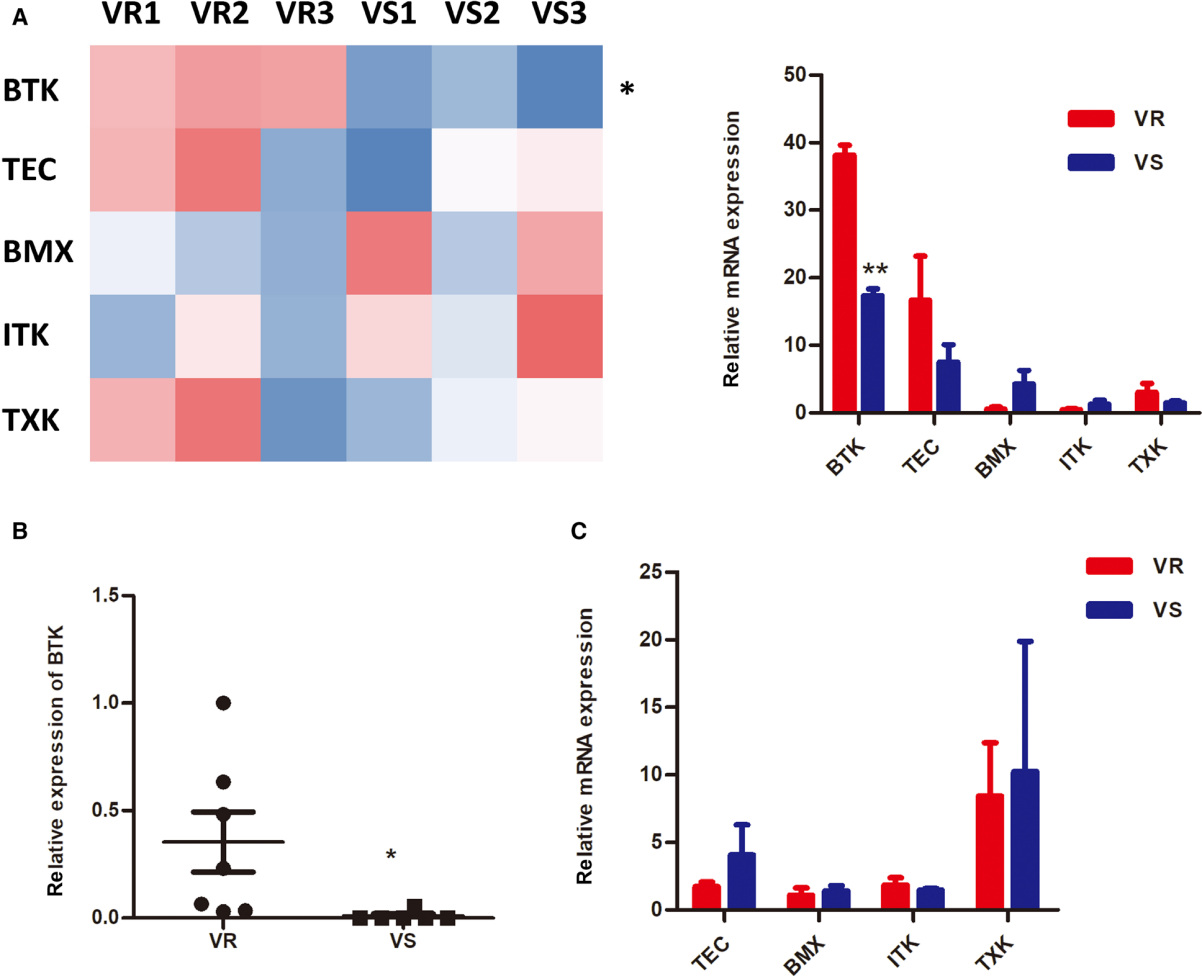
(A) RNA sequencing of bone marrow mononuclear cells from three patients who did not achieve remission with venetoclax‐based treatment and three cases of complete remission. The differential expression of genes in the tyrosine kinase family was determined. VR1‐3 (red) shows higher expression of BTK versus VS1‐3 (blue). Data are plotted as mean ± standard error of the mean (SEM) (*N* = 3 in each group). (B) The relative expression of BTK in BM of seven cases of nonremission and six cases of complete remission in AML patients was measured by quantitative PCR. Data are plotted as mean ± SEM (*N* = 7 and 6). (C) The relative expression of TEC, BMX, ITK, and TXK in BM of seven cases of nonremission and six cases of complete remission in AML patients was measured by quantitative PCR. Data are plotted as mean ± SEM (*N* = 7 and 6).* for *P* < 0.05 and ** for *P* < 0.01.

To explore the potential mechanisms of resistance to venetoclax in AML patients, we applied paired RNA sequencing of the bone marrow mononuclear cells of six patients who received venetoclax treatment, three of whom went into complete remission and three who did not. The results demonstrated that there were multiple gene expression differences between the two sets of samples, and we noticed a significant difference in the expression levels of BTK (Fig. 1A). To further confirm the sequencing results, we expanded the sample size and used quantitative PCR to detect the expression of BTK (Fig. 1B) and its other family members (Fig. 1C) in bone marrow mononuclear cells of seven AML patients resistant to venetoclax and six who were sensitive to it. Interestingly, we showed similar results to the sequencing, which indicated that AML patients who develop venetoclax resistance might appear to have higher expression of BTK.

### 3.2 Abivertinib and venetoclax have a synergistic effect on AML cell lines without harming human CD34‐positive hematopoietic stem cells

**Fig. 2:**
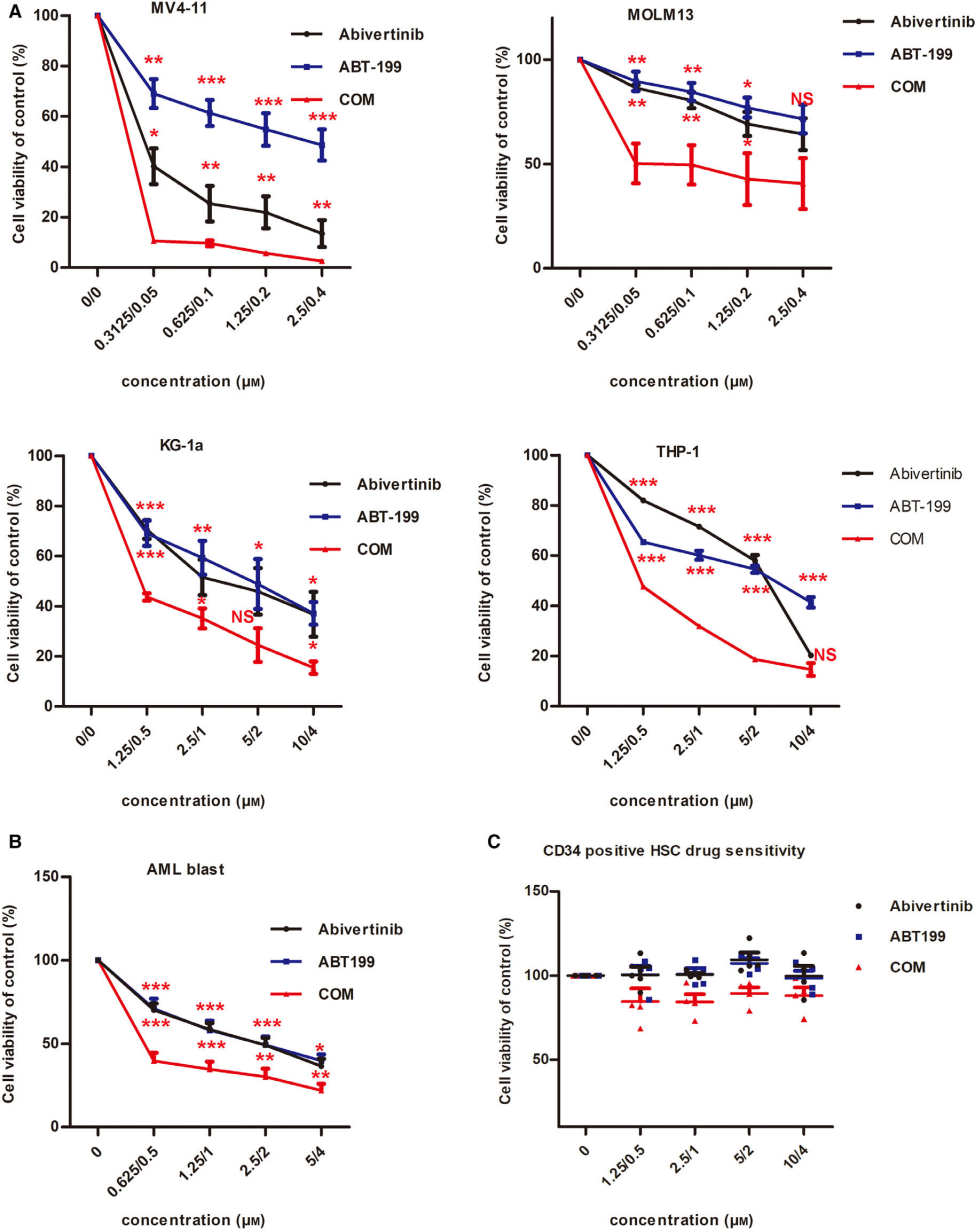
(A) Changes in the cell viability of AML cell lines (MV4‐11, MOLM‐13, KG‐1a, THP‐1) treated with abivertinib (0.3125, 0.625, 1.25, and 2.5 μm in MV4‐11/MOLM13 and 1.25, 2.5, 5, and 10 μm in KG‐1a/THP‐1), venetoclax (0.05, 0.1, 0.2, and 0.4 μm in MV4‐11/MOLM13 and 0.5, 1, 2, and 4 μm in KG‐1a/THP‐1), and COM. Data are plotted as mean ± standard deviation (SD) (*N* = 3). (B) Cell viability of AML blasts treated with abivertinib, venetoclax, and COM. Data are plotted as mean ± SD (*N* = 12). (C) Change in the cell viability of healthy CD34‐positive cord HSC treated with abivertinib, venetoclax, and COM. Data are plotted as mean ± SD (*N* = 4). * for *P* < 0.05, ** for *P* < 0.01, and *** for *P* < 0.001.

According to the results above, we were inspired to treat AML cells with BTK inhibitors and BCL‐2 inhibitors synchronously. We investigated the proliferation inhibitory effects of increasing concentrations of abivertinib and venetoclax alone or in combination on AML cell lines. As shown in Fig. 2A, the inhibition of cell proliferation in the combination group was significantly greater than that in either of the two monotherapy groups. The combination index and curve are shown in Table S1 and Fig. S1. This result preliminarily demonstrated the synergistic effect of abivertinib and venetoclax in AML cell lines. Next, in order to further explore whether there is a similar synergistic effect on primary AML cells, we treated 12 samples of mononuclear cells from the peripheral blood or bone marrow of AML patients (AML3# and AML12# venetoclax‐resistant), including those bearing various genetic abnormalities, with a combination of abivertinib and venetoclax or with single agents. The results are shown in Fig. 2B and indicated that abivertinib and venetoclax also have a significant synergistic effect on primary AML cells. More interestingly, according to the results, the combination of abivertinib with venetoclax can benefit patients who are resistant to venetoclax.

At the same time, in order to evaluate the safety of the combined use of the two drugs, we treated the CD34‐positive hematopoietic stem cells derived from healthy cord blood with these two drugs. As shown in Fig. 2C, there was almost no harmful effect on normal healthy cells.

### 3.3 Abivertinib combined with venetoclax affects the proliferation of AML cells

**Fig. 3:**
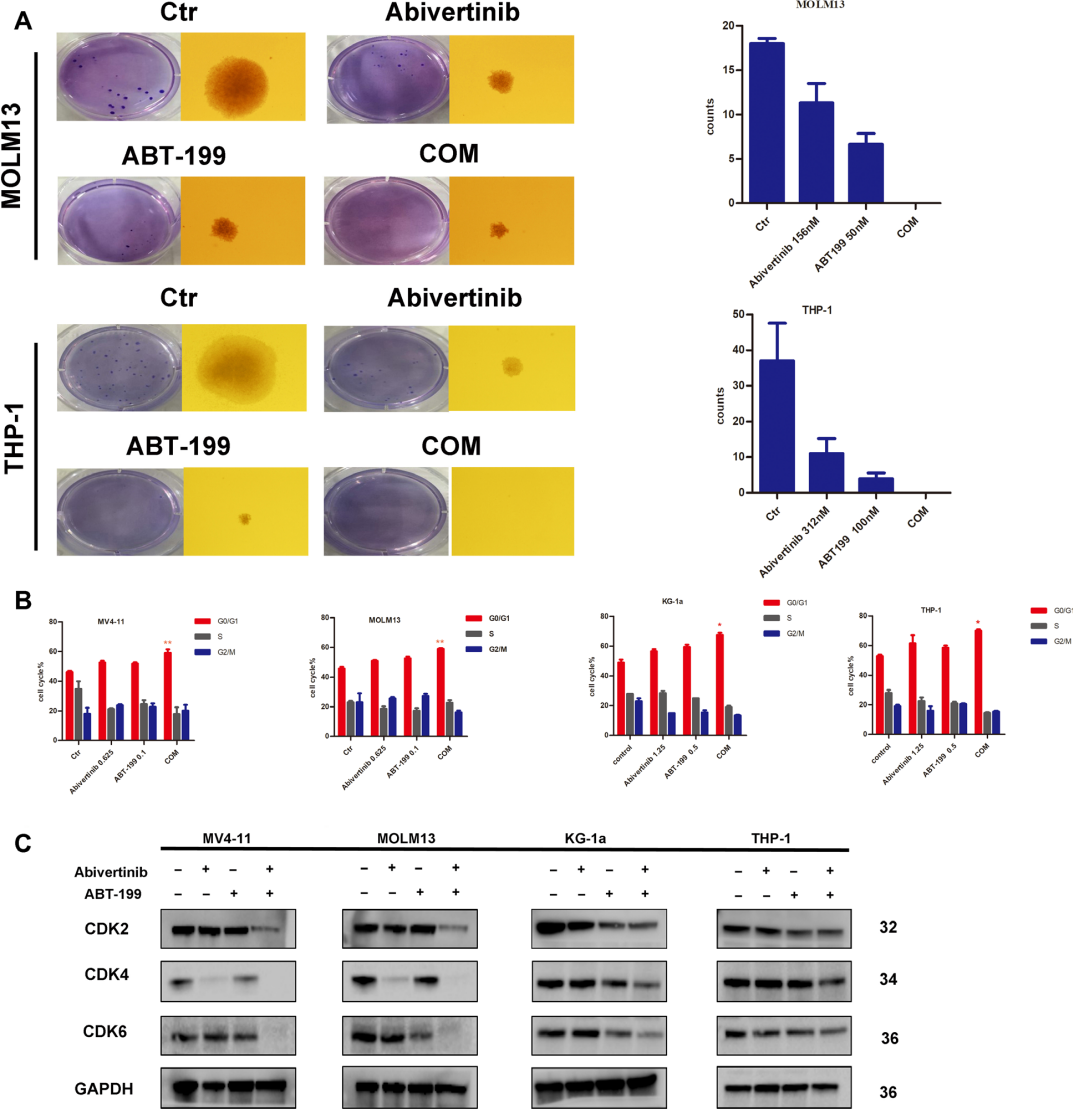
(A) Clone formation of AML cell lines treated with abivertinib (156 nm for MV4‐11 and 325 nm for THP‐1), venetoclax (50 nm for MV4‐11 and 100 nm for THP‐1), and COM. Data are plotted as mean ± standard deviation (SD) (*N* = 3). (B) Cell cycle distribution of AML cell lines (MV4‐11, MOLM‐13, KG‐1a, and THP‐1) treated with abivertinib, venetoclax, and COM. Data are plotted as mean ± SD (*N* = 3). (C) The protein levels of cyclically dependent proteins CDK2, CDK4, and CDK6 were determined by western blot. * for *P* < 0.05, ** for *P* < 0.01, and *** for *P* < 0.001.

To investigate the efficacy of abivertinib combined with venetoclax on the proliferation of AML cells, we first evaluated their effect on AML clone formation ability using a soft agar colony formation assay. As shown in Fig. 3A, both drugs alone could decrease the colony numbers and size of MOLM‐13 and THP‐1, which demonstrated that they inhibit the clone‐forming capacity of AML cells. Even more remarkable, when the two drugs are used in combination, AML cells almost lose the ability to develop clones.

Next, we exposed AML cells to abivertinib, venetoclax, or their combination for 24 h followed by flow cytometry detection of cell cycle distribution. The results in Fig. 3B show that the combination of the two drugs can block the progression of the AML cell cycle in the G0/G1 phase. In the assessment of the expression levels of the cycle‐dependent protein family at the G0/G1 phase, the CDK2, CDK4, and CDK6 proteins were observed to be downregulated (Fig. 3C), which caused the cell cycle to stagnate and fail to enter DNA synthesis, thereby killing the AML cells.

**Fig. 4:**
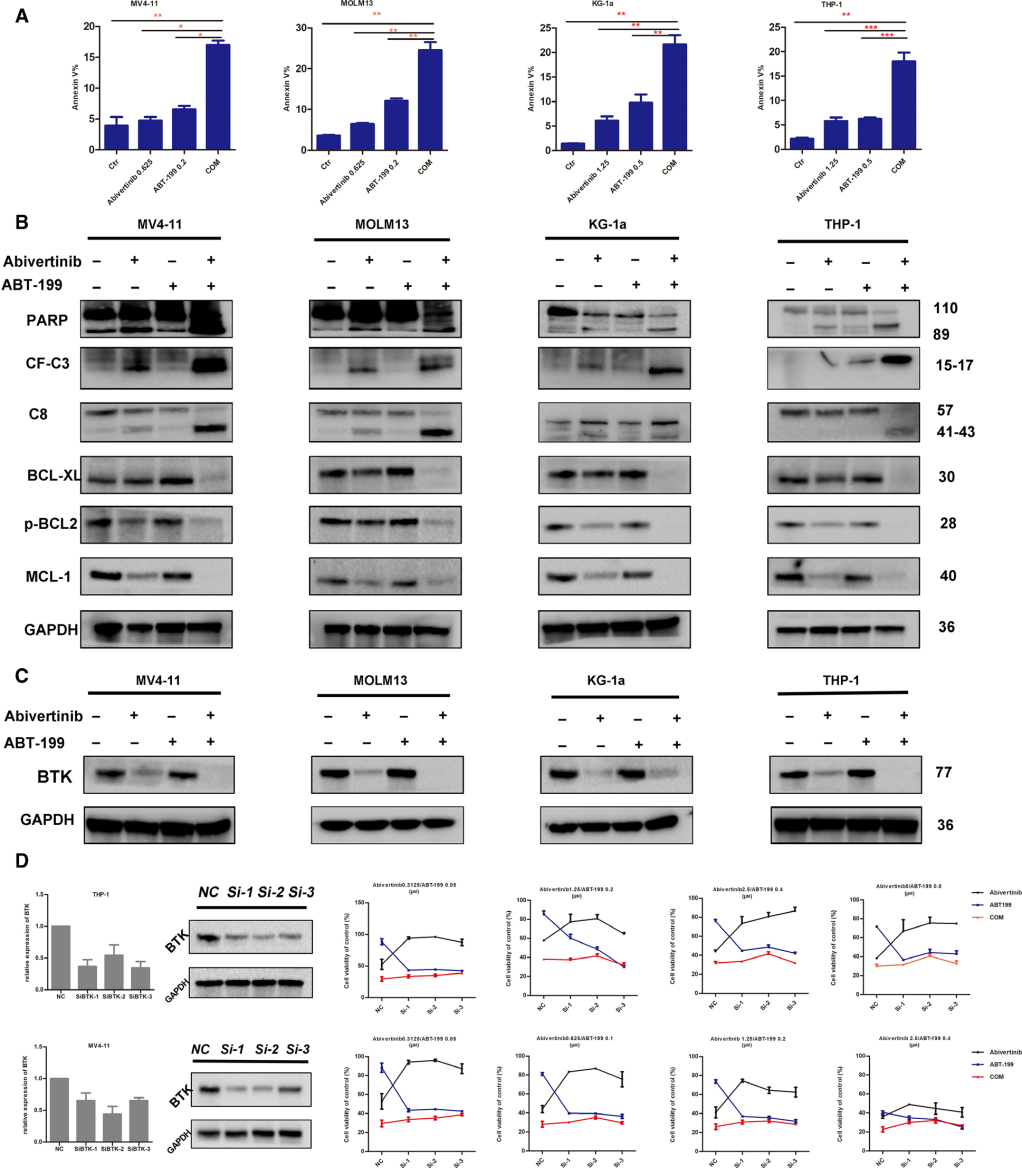
Apoptotic AML cells induced by abivertinib, venetoclax, and COM. Data are plotted as mean ± standard deviation (SD) (*N* = 3). (B) The levels of caspase family proteins (CF‐C3, PARP, and C8) and BH3 family proteins (BCL‐XL, MCL‐1, and p‐BCL‐2) in AML cells after drug exposure were determined by western blot. The same doses were used in both experiments. (C) The protein level of BTK in AML cells after different treatments was measured. (D) Knockdown of BTK in THP‐1 and MV4‐11 by siRNA technology and the cell viability of AML cells with or without knockdown of BTK treated with abivertinib, venetoclax, and COM. Data are plotted as mean ± SD (*N* = 3).

Considering that venetoclax is an inhibitor of anti‐apoptotic protein BCL‐2 that targets the apoptotic pathway, we next focused on whether the combination of the two drugs could cause a synergistic induction of apoptosis. First, we used flow cytometry to detect the proportion of apoptotic cells among AML cells treated with single‐agent or combination therapy. The results (Fig. 4A) showed that the combination of the two drugs could induce significant cell apoptosis compared with that of any other single drug. Upon further exploration of the mechanism of apoptosis, we detected the synergistic activation of caspase family proteins in the exogenous apoptotic pathway, including the emergence of cleaved bands of PARP, caspase‐8, and caspase‐3 proteins in the terminal pathway (Fig. 4B). These results suggested that the combination of the two drugs could activate the exogenous apoptotic pathway to induce AML cell apoptosis. Of course, we also tested the expression of anti‐apoptotic proteins in the BH3 family. The results showed that the expression of BCL‐XL and MCL‐1 was significantly decreased with combination therapy, while the apoptotic protein MCL‐1 was slightly upregulated when venetoclax was used alone. Our results indicate that BTK inhibitors can downregulate other proteins in the BH3 family after venetoclax exposure, thereby improving their combined efficacy. In addition, we also tested the expression of p‐BCL‐2 (Fig. 4B); both drugs can downregulate its expression, but the effect of the combination of two drugs is more obvious compared with those of the single agents. Therefore, we could conclude that abivertinib combined with venetoclax suppress AML cell survival by inhibiting cell clone formation, arresting cell cycle, and inducing cell apoptosis.

### 3.4 Abivertinib and venetoclax synergistically restrain AML progression in a BTK‐dependent manner

To further explore the mechanism of synergistic function between abivertinib and venetoclax, we measured the protein expression levels of BTK (Fig. 4C) and its downstream proteins (Fig. S3) in AML cell lines in different treatment groups. The results demonstrated significant downregulation of the BTK pathway in the combined group and a slight upregulation in the group with venetoclax alone. This result is very similar to that of the apoptotic family protein MCL‐1.

We hypothesized that the synergistic effect of these two drugs is associated with the ability of abivertinib to downregulate the expression of BTK or MCL‐1, which are upregulated by venetoclax treatment. To confirm that, we knocked down BTK and MCL‐1 in AML cells (THP‐1 and MV4‐11) by siRNA technology as shown in Fig. 4D,E and Fig. S4A,B. Then, we incubated AML cells with different expression levels of BTK or MCL‐1 with abivertinib, venetoclax, or their combination followed by cell proliferation assay evaluation. According to the results, when we knocked down BTK in THP‐1 and MV4‐11, the combination effect between abivertinib and venetoclax was no longer seen. However, after knocking down MCL‐1 in AML cells, the result was the exact opposite: There was still a good synergistic effect. These results indicated that the synergistic effect of abivertinib and venetoclax occurs through a BTK‐dependent manner rather than acting through MCL‐1.

### 3.5 Abivertinib combined with venetoclax reduces AML xenograft tumor burden and prolongs host survival

**Fig. 5:**
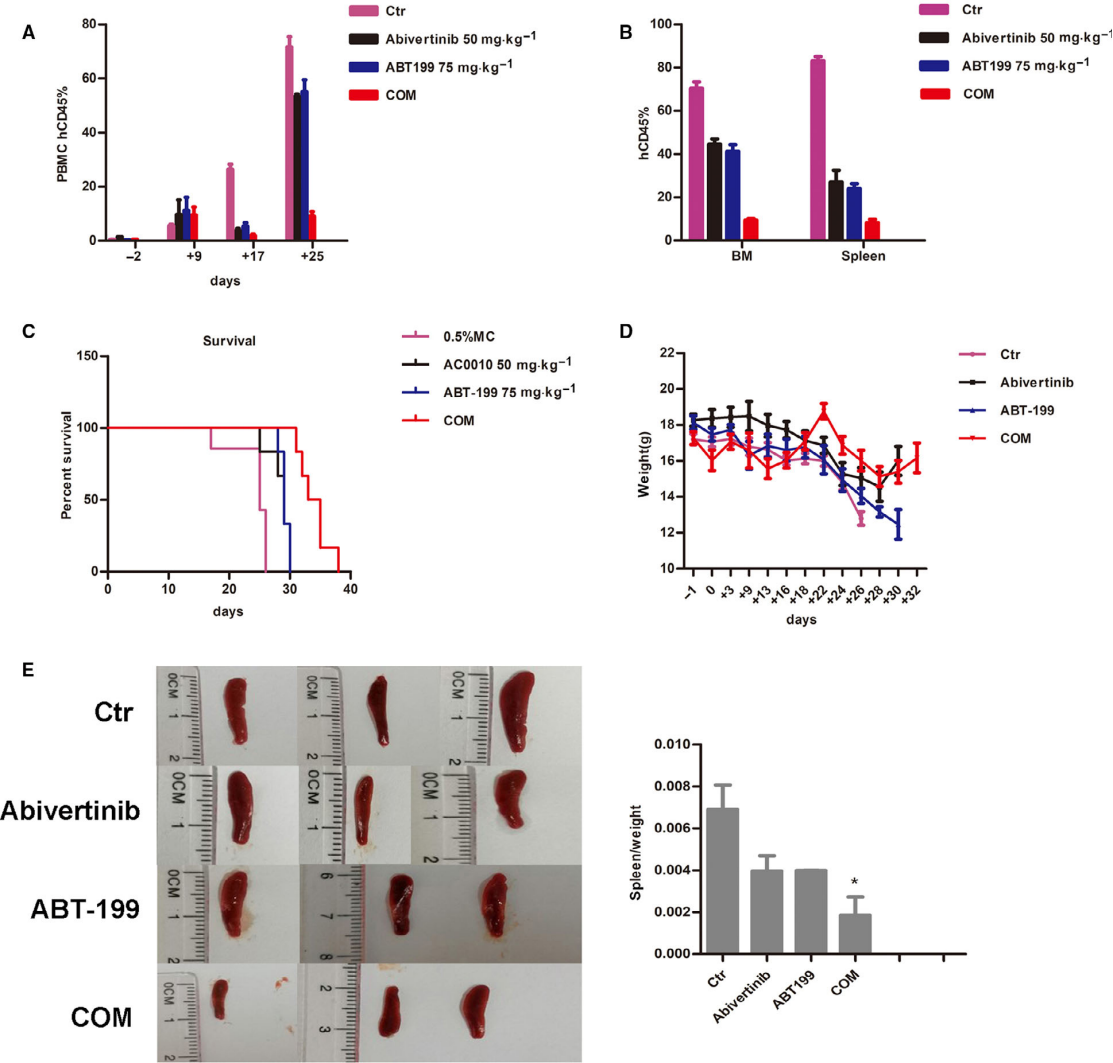
The antileukemia effect of abivertinib plus venetoclax in MV4‐11 xenograft mice. (A) Leukemia tumor burden assessed by human CD45 expression in PBMCs following treatment for up to 21 days or until sacrifice. Data are presented as mean ± SEM in each group (*N* = 6) (B) Kaplan–Meier survival time (time to hemiplegic paralysis) for the duration of the treatment. (C) Body weight change versus time. Data are presented as mean ± SEM in each group (*N* = 6). (D) Percentages of human CD45 in the bone marrow and spleen of mice from different treatment groups after sacrifice. (E) Ratio of spleen to total body weight measurements from the abovementioned experiments. Data are presented as mean ± SEM in each group (*N* = 3). * for *P* < 0.05, ** for *P* < 0.01, and *** for *P* < 0.001.

The effects of abivertinib and venetoclax *in vivo* were investigated in MV4‐11‐NSG xenograft mice. All the drug administrations referred to started 7 days after injection of MV4‐11 cells by tail vein. All mice had equal tumor burdens, measured by testing for human CD45 in PBMCs at the beginning of therapy. In the following days, we tested the levels of human CD45 PBMCs of the mice in each group every week to evaluate the tumor burden of different treatment groups. Mice treated with a combination of abivertinib (50 mg·kg^−1^) and venetoclax (75 mg·kg^−1^) had a significantly lower leukemia burden during the treatment period compared with that of those treated with vehicle or only a single drug (*P* < 0.05; Fig. 5A). Mouse survival time was marked by the appearance of paralysis in the lower limbs as the end event. AML mice in both the monotherapy and the combination groups had significantly prolonged survival times, but the combined group did significantly better than any single‐drug group (*P* < 0.0001). The median survival time of the vehicle group was 25 days, and the abivertinib and venetoclax single‐drug groups were 28.5 and 29 days, respectively, while the combined group was 34 days (Fig. 5B).

During the study, we also recorded the body weight of the mice in each group every two days to assess treatment tolerance. There was no significant difference among the four groups, which indicated our drug administration did no harm to the mice (Fig. 5C).

When the mice were humanely sacrificed, samples were collected to detect the expression level of human CD45 by flow cytometry and immunohistochemistry. According to the results from flow (Fig. 5D), both agents alone could decrease the expression level of human CD45, while coadministration of abivertinib and venetoclax resulted in the lowest level of engraftment. In addition, we measured the weight of the mouse spleens and then calculated the spleen‐to‐body weight ratio (Fig. 5E). The results also showed that the combined group had the smallest ratio. Similar results were observed in the following immunohistochemistry of bone marrow and spleen (Fig. S5A,B). All our results demonstrated that abivertinib synergized with venetoclax to reduce AML xenograft tumor burden.

## 4 Discussion

In this study, the synergistic antileukemic activity of abivertinib and venetoclax was observed *in vitro* and *in vivo*, and *in vitro* results indicated that this combination has the potential to improve outcomes in AML patients who are resistant to venetoclax‐based therapy. As far as we know, this is the first study investigating the potential synergy of two oral agents, abivertinib and venetoclax, in AML. Considering the clinical feasibility of abivertinib and venetoclax, we believe that the combination of these two drugs can benefit elderly AML patients who cannot tolerate chemotherapy.

The application of venetoclax in AML is now quite extensive. The clinical trial data of combined demethylating drugs, such as azacitidine, also reveal a good remission rate. However, many patients still cannot reach remission, or they develop resistance and recurrence in a short time. Recently, more and more research focuses on the upregulation of other anti‐apoptotic proteins of the BH3 family, especially MCL‐1, after venetoclax treatment. A number of reports have also indicated that high expression of MCL‐1 is responsible for nonremission following venetoclax treatment, and combined treatment with an MCL‐1 inhibitor can reverse the original resistance in AML cells. In our study, we identified biomarkers that may be clinically responsible for resistance to venetoclax treatment from paired RNA‐seq results. The expression of tyrosine kinase BTK in patients with no remission was significantly higher than that in those with remission. In addition, we showed the differential expression of other family members of TEC (TEC, BMX, TXK, ITK), but the difference was not significant (Fig. 1A). Next, we expanded the sample size to verify that the expression level of BTK was indeed significantly higher in nonremission or drug‐resistant patients than remission patients. This result was also confirmed in AML cell lines treated with venetoclax, which showed a slight upregulation of BTK (Fig. 4C). For other TEC family members shown in Fig. 1C, the difference in expression levels of TEC, TXK, and ITK between two groups of patients was not significant. Although the low expression of BMX was detected, further research is planned. On the other hand, we also examined the expression change in MCL‐1. Surprisingly, neither the small sample RNA sequencing nor the postamplification verification yielded results consistent with the previous report (Fig. S2A,B). However, a slight upregulation of MCL‐1 was detected in an AML cell line treated with venetoclax (Fig. 4B). Due to the limited number of samples used for RNA sequencing, we need to acquire more primary patient samples to confirm our results in the future.

In recent years, in order to rescue the clinical failure of venetoclax treatment in AML patients, many medical centers have carried out clinical trials involving in venetoclax‐based therapy, particularly when combined with azacitidine. Fortunately, most of them have achieved very good results; however, some patients are still unable to achieve remission. Even more troubling is that the mechanism behind the effectiveness of this treatment regimen is unclear, and basic research in this area is still lacking. Therefore, our study suggests that a dual oral regimen may lead to a promising chemo‐free therapy for those patients.

In our study, we treated AML with BTK inhibitor in combination with venetoclax based on our preliminary results. We picked a novel BTK inhibitor, abivertinib, which is currently undergoing phase II clinical trials for lymphoma in our center. In a study by Xu *et al*. [29], they showed abivertinib exhibited greater than 80% inhibition in 33 of 349 unique kinase assays including JAK3, BTK, and 5 TEC family members at a concentration of 1 mmol·L^−1^. Moreover, abivertinib had a well‐tolerated safety profile and promising antitumor activity in patients with NSCLC with acquired resistance to a first‐generation EGFR TKI, supporting its continued development [30]. Our group has also previously demonstrated in early research that abivertinib has a significant antileukemia effect [28, 31]. In this study, we demonstrated abivertinib could have a good synergistic effect with venetoclax in inhibiting AML cell proliferation. In terms of AML patients, there were 12 patients, including 3 cases of relapse or refractory (two of them did not carry out remission treated with venetoclax and azacitidine) and 9 cases in initial treatment (one case carrying *FLT3‐ITD* mutation, one case carrying *DMNT3A* mutation), sensitive to the combination of abivertinib and venetoclax. The combined index ED50 < 1 also indicated a synergistic effect. Our results in cell lines and AML blasts indicate that the combination treatment shows a potential inhibitory effect in a wide‐type manner, whether they are carrying the *FLT3‐ITD* mutation or are hard to deal with in clinical practice. Since the safety of the two drugs alone has been clinically recognized, our study simultaneously evaluated both drugs in CD34‐positive healthy cord blood stem cells and found no significant damage. Presumably, this also paves the way for the clinical implementation of this treatment.

In the existing research, many scholars have also tried other drugs combined with venetoclax to treat preclinical AML. For example, Professor Michael R. Savona focused on the apoptosis family pathway (plus some others) to illustrate the synergistic effect of an MCL‐1 inhibitor and venetoclax [1]. For cyclin protein inhibitor combination, the author also highlighted MCL‐1 [17]. In this study, we also explored the potential mechanisms by which abivertinib and venetoclax synergistically suppress AML progression. In our earlier studies, we showed that abivertinib has a wide range of antileukemia effects, so we have done more exploration of the joint mechanism, which is different in the reported research. We first observed the effects of the combination on the biological functions of AML, such as clone forming, apoptosis, and cell cycle arrest. The results indicated that the two could synergistically inhibit the ability of AML clone formation. In terms of cell cycle, the cell division stage of the combined treatment group was significantly blocked in the G0/G1 phase, thus, the DNA synthesis phase could not be performed, and AML cell stop proliferation. In the induction of apoptosis, we focused on the mitochondrial anti‐apoptotic protein family and the exogenous caspase family. Western blot results showed that p‐BCL‐2, BCL‐XL, and MCL‐1 anti‐apoptotic proteins were downregulated in the drug cotreatment group. Additionally, the appearance of caspase‐3, caspase‐8, and PARP cleavage bands in the combination group was also detected. All of these results indicated that abivertinib and venetoclax could synergistically induce apoptosis in AML cells.

Next, in order to verify whether the combined effect of the two agents depends on BTK, we performed a drug sensitivity test on the AML cell lines with or without BTK knockdown, and the results showed that the synergistic effect of the two drugs diminished in the AML cells with impaired BTK expression. Because of this, we proposed that the synergy between abivertinib and venetoclax in AML is dependent on the level of BTK. When BTK expression is downregulated, abivertinib fails to synergize with venetoclax to suppress AML cells. In addition, it is worth mentioning that the sensitivity of AML cells to venetoclax increases after downregulation of BTK, which may be the reason for the disappearance of the joint effect. Therefore, in order to rule out the important role of MCL‐1 in the combined action of these two drugs, we also performed MCL‐1 knockdown followed by drug susceptibility tests on AML cells, but the combined effect of the two did not change significantly. Therefore, in this study, we confirmed that abivertinib synergized with venetoclax to block AML progression in a BTK‐dependent manner.

In our study, *in vivo* experiments have also been initially explored. According to our results, the combination of abivertinib and venetoclax can significantly prolong the survival time of MV4‐11 mice and reduce the tumor burden at the same time. Even more interestingly, the antileukemia effect in the combination group is much better than that in any single‐drug treatment group. Similar results were observed in THP‐1‐luci‐NSG xenograft mice. The results showed in Fig. S6 demonstrated that cotreatment with abivertinib and venetoclax could synergistically reduce the tumor burden of AML mice. Moreover, based on the changes in body weight of mice in each group, we claimed that the drug cotreatment could be tolerated. However, we know that data from the more relevant PDX leukemia mouse model are more valuable for clinical use, but our research group currently lacks the experience to utilize this model, which is the direction of our follow‐up efforts. Nevertheless, the results of this *in vivo* experiment can also explain the significant antileukemia effect of the combination of the two drugs, and we are confident in their use for the clinical treatment of AML patients who do not respond to venetoclax treatment.

## 5 Conclusion

Our results showed that the combination of abivertinib and venetoclax can inhibit the progression of AML *in vivo* and *in vitro*, especially in patients who did not achieve remission with venetoclax‐involved treatment.

## Conflict of interest

The authors declare no conflict of interest.

## Author contributions

SH conceived and designed the study, performed the experiments, analyzed the data, and wrote the manuscript; CL, XZ, YL, JP, FL, JH, QL, WY, SM, and XH performed the experiments, analyzed the data, and participated in the manuscript preparation; and JJ supervised the study, contributed to the design of experiments, analyzed the data, and revised the manuscript.

## Ethics approval and consent to participate

Our animal study was approved by the Ethics Committee for Laboratory Animals of the First Affiliated Hospital, College of Medicine, Zhejiang University (Hangzhou, China), and was conducted in accordance with the National Institutes of Health Guide for the Care and Use of Laboratory Animals.

## Supporting information


**Fig. S1.** The combination curve of AML cells treated by abivertinib and venetoclax.
**Fig. S2.** The expression of MCL1 in AML patients treated with ‘VA’ therapy.
**Fig. S3.** The protein level of PLCγ2，ERK and NF‐κB at downstream of BTK signal was measured after different treatments in AML cells.
**Fig. S4.** The effect of MCL‐1 impaired expression to the combination of abivertinib and venetoclax.
**Fig. S5.** IHC of femurs and spleen of mice from different groups.
**Fig. S6.** Leukemia burden of THP‐1‐luciferase AML mice in different treatment groups.
**Table S1.** The combination index of AML cells treated by abivertinib and venetoclax.
