# Supplementary material for: Abivertinib synergistically strengthens the anti‐leukemia activity of venetoclax in acute myeloid leukemia in a BTK‐dependent manner
Source: Mol Oncol. 2020 Jul 3;14(10):2560–73. doi: 10.1002/1878-0261.12742 (PMC7530784; doi:10.1002/1878-0261.12742)
Supplement: Supplementary file 1 — Fig. S1. The combination curve of AML cells treated by abivertinib and venetoclax. Fig. S2. The expression of MCL1 in AML patients treated with ‘VA’ therapy. Fig. S3. The protein level of PLCγ2，ERK and NF‐κB at downstream of BTK signal was measured after different treatments in AML cells. Fig. S4. The effect of MCL‐1 impaired expression to the combination of abivertinib and venetoclax. Fig. S5. IHC of femurs and spleen of mice from different groups. Fig. S6. Leukemia burden of THP‐1‐luciferase AML mice in different treatment groups. Table S1. The combination index of AML cells treated by abivertinib and venetoclax. [file MOL2-14-2560-s001.docx]

Abivertinib synergistically strengthens the anti-leukemia activity of venetoclax in acute myeloid leukemia in a BTK-dependent manner

Running title: Abivertinib could rescue AML insensitive to vetenoclax-involved therapy

Shujuan Huang^1,2^, Chenying Li^1,2^, Xiang Zhang^1,2^, Jiajia Pan^1,2^, Fenglin Li^1,2^, Yunfei Lv^1,2^,Jingwen Huang^1,2^, Qing Ling^1,2^, Wenle Ye^1,2^, Shihui Mao^1,2^, Xin Huang^1,2^, Jie Jin^1,2^.

1 Department of Hematology, the First Affiliated Hospital, Zhejiang University College of Medicine, Hangzhou, People’s Republic of China

2 Key Laboratory of Hematologic Malignancies, Diagnosis and Treatment, Zhejiang, Hangzhou, People’s Republic of China

**Corresponding author**: Prof. Jie Jin

Department of Hematology, the First Affiliated Hospital, Zhejiang University School of Medicine, No.79 Qingchun Road, Hangzhou 310003 Zhejiang, P.R. China.

E-mail: [jiej0503@zju.edu.cn](mailto:jiej0503@zju.edu.cn)

Telephone number:18143465818

Table1. AML cells combination index

Sample ED50 ED75 ED90

MV4-11 0.04 0.06 0.08

MOLM13 0.8 0.44 0.3

THP-1 0.5 0.4 0.4

KG-1a 0.4 0.56 0.85

1# 0.26889 0.18038 0.43562

2# 0.25572 1.06235 6.67873

3# 0.68133 0.58564 0.56388

4# 0.06217 0.58852 16.20546

5# 0.71855 0.66073 0.62357

6# 5.2824e-005 0.16814 5829.65157

7# 1.20843 3.29632 10.61602

8# 0.06217 0.58852 16.20546

9# 0.07606 1.08113 15.90189

10# 0.17971 0.25471 0.39547

11# 0.33678 0.56422 1.09086

12# 0.60859 0.76234 1.85561

AML12#, ABT-199 resistance, DNMT3A R8882H (+) 45%, FLT3-ITD (+) 39%, NPM1 (+)

AML7#, PML-RARA mutation (+)

AML3#, 12# ABT-199 resistance


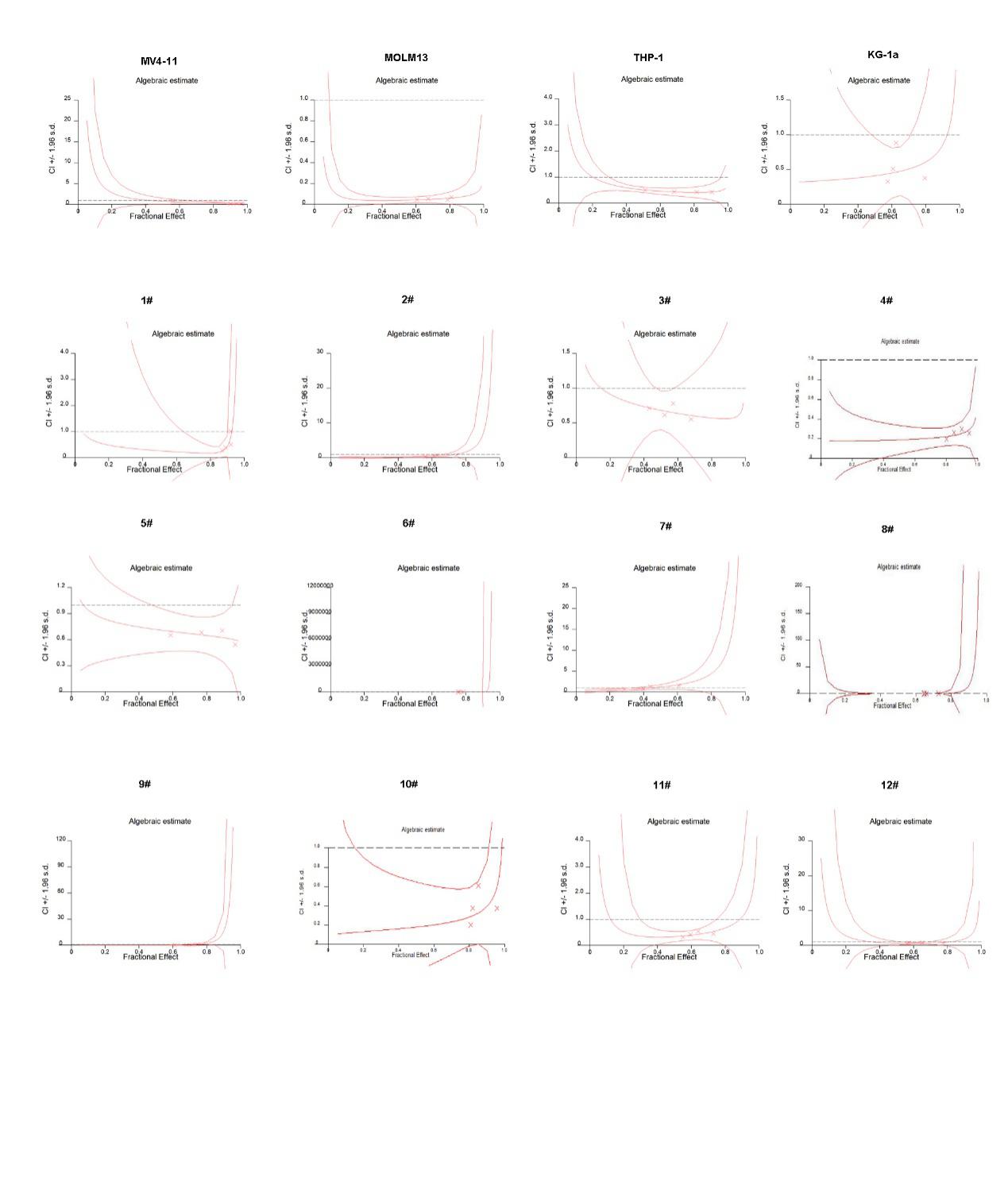
Fig1：The combination curve of AML cells treated by abivertinib and venetoclax.


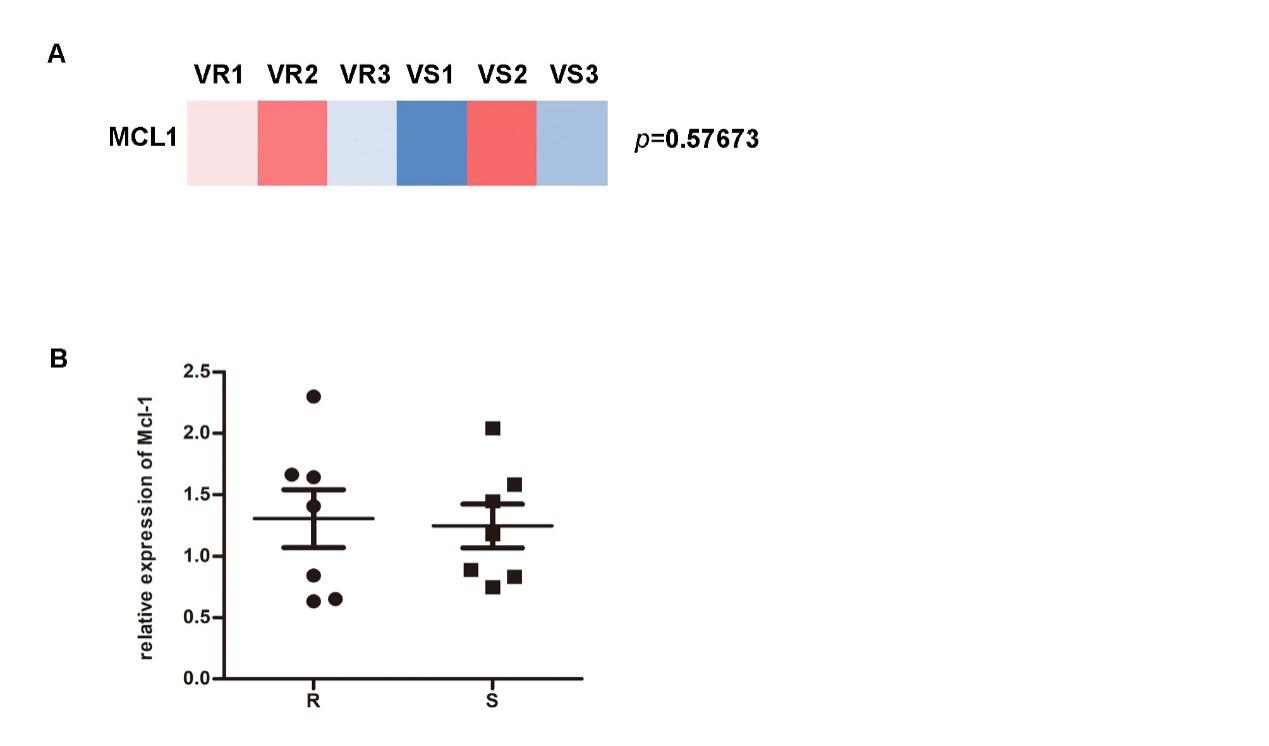


Fig2: **(A)** RNA-sequencing of bone marrow mononuclear cells from three cases of patients who did not achieve remission with venetoclax -based treatment and three cases of complete remission. The differential expression of genes in tyrosine kinase family was determined.VR1-3(red) show higher expression of MCL1 versus VS1-3(blue). (**B)** The relative expression of MCL1 in BM of 6 cases of non-remission and 5 cases of complete remission AML patients was measured by quantitative PCR method bone.


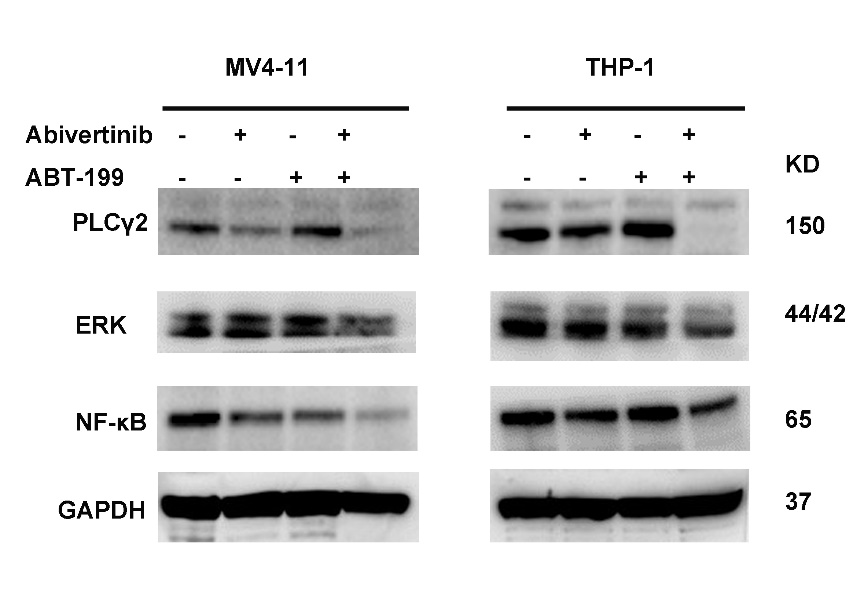


Fig3: The protein level of PLCγ2，ERK and NF-κB at downstream of BTK signal was measured after different treatment in AML cells.


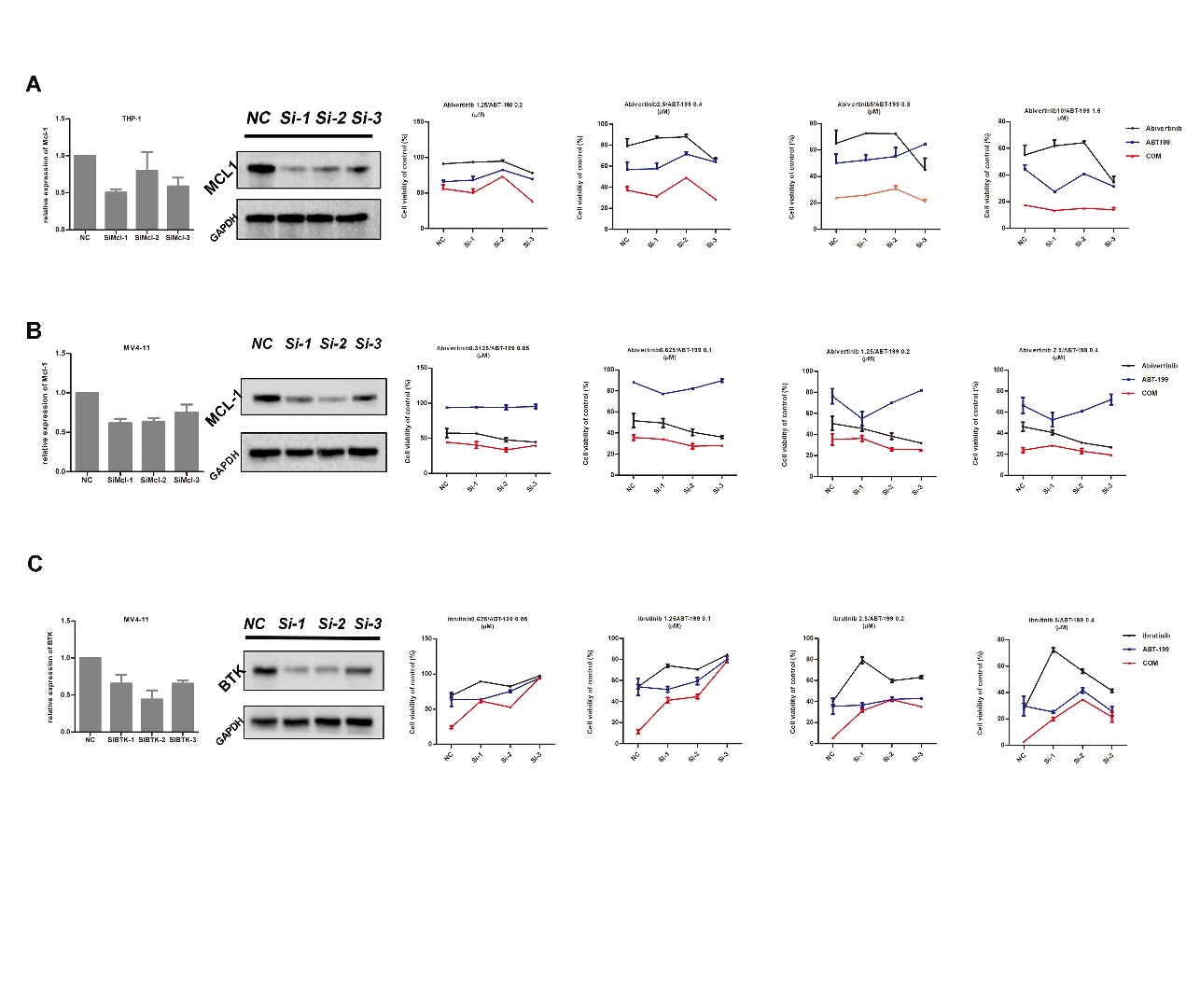


Fig4 (**A)** Knock down of MCL-1 in THP-1 by SiRNA technology and the cell viability of AML cells with or without knock down of MCL-1 treated Abivertinib, venetoclax, and COM. **(B)** Knock down of MCL-1 in MV4-11 by SiRNA technology and the cell viability of AML cells with or without knock down of MCL-1 treated Abivertinib, venetoclax, and COM. **(C)** Knock down of BTK in MV4-11 by SiRNA technology and the cell viability of AML cells with or without knock down of BTK treated ibrutinib, venetoclax, and COM.


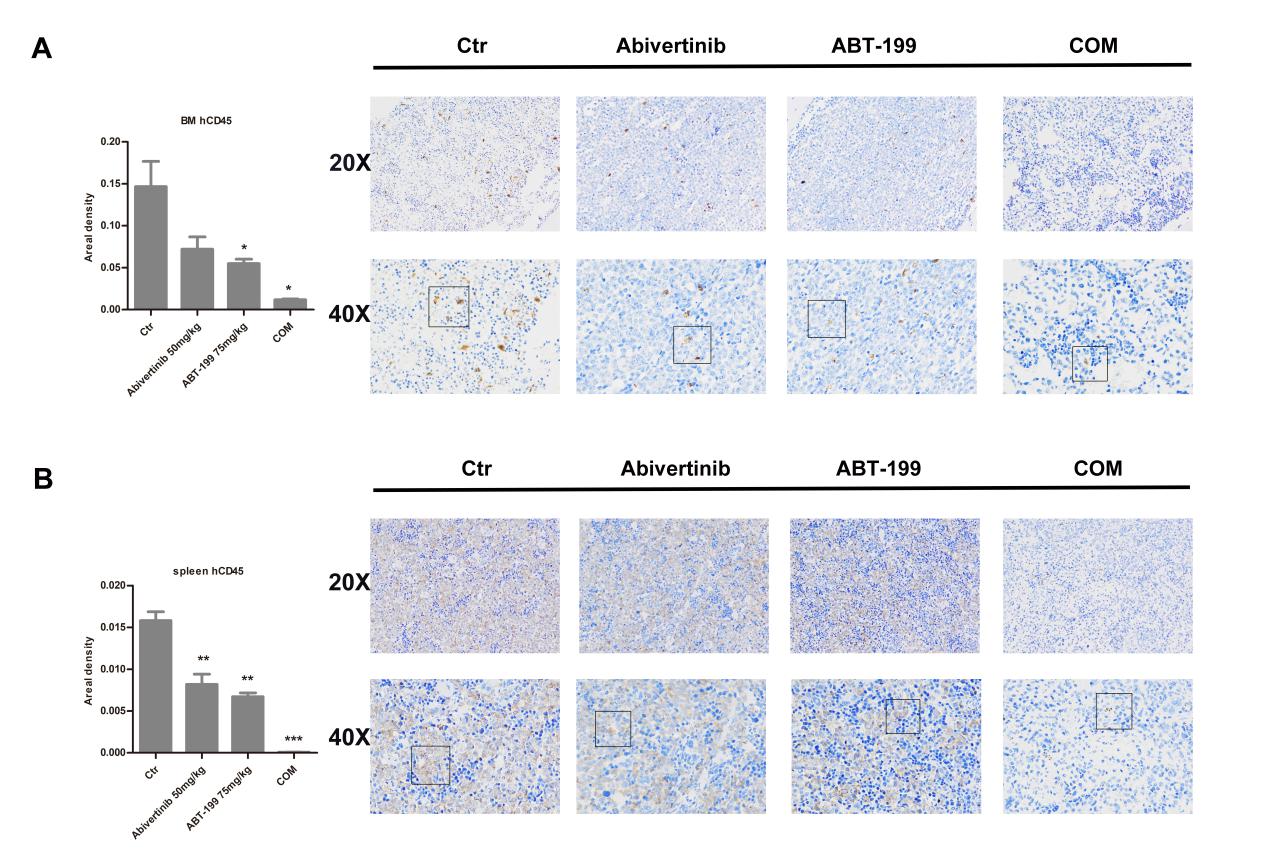


Fig5: IHC of femurs (A) and spleen (B) (20× and 40×) stained with monoclonal antibody for hCD45 reveal AML cells left within the bone marrow and spleen of experimental mice at different group. * for p<0.05, ** for p<0.01, *** for p<0.001.


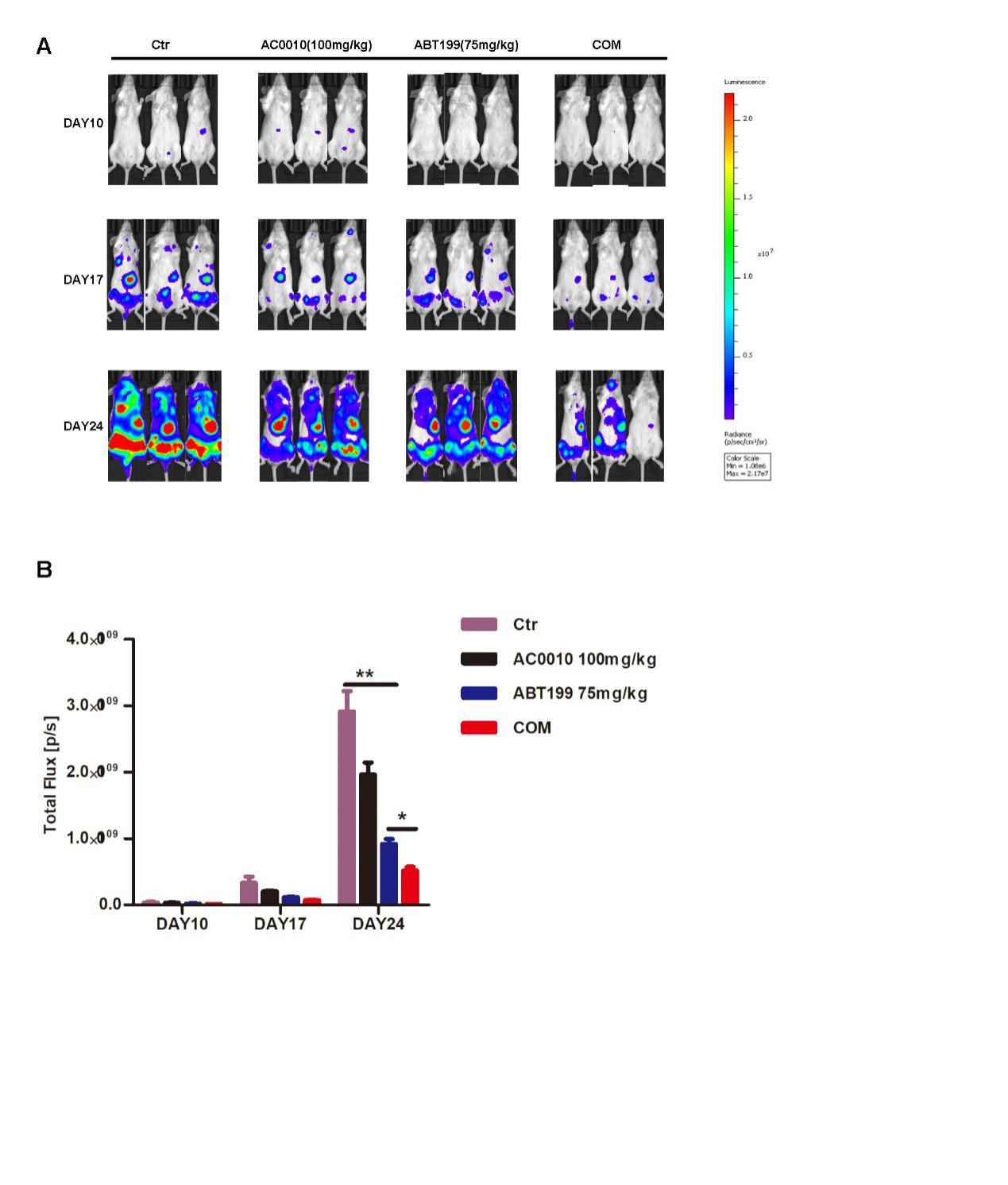


Fig6: (A) and (B) Bioluminescence measurements of photon intensity showing relative leukemia burden following injection of THP-1-luci cells up to 24 days.
